# 1187. Evaluation of an Antimicrobial Stewardship (AS) Bundle for Uncomplicated Gram-Negative Bloodstream Infections (GN-BSI) in Adults at a Large Academic Health System

**DOI:** 10.1093/ofid/ofad500.1027

**Published:** 2023-11-27

**Authors:** Juliana M Dipietro, Yanina Dubrovskaya, Kassandra Marsh, Arnold Decano, Dana Mazo, Vincent J Major, Jonathan So, Samuel Yuditskiy, John Papadopoulos, Justin Siegfried

**Affiliations:** Flushing Hospital medical Center, Secaucus, New Jersey; NYU Langone Health, New York, New York; NYU Langone Health, New York, New York; NYU Langone Health, New York, New York; New York University, New York University, NY; NYULH, New York, New York; NYULH, New York, New York; NYULH, New York, New York; NYU Langone Health, New York, New York; NYU Langone Health, New York, New York

## Abstract

**Background:**

AS bundles provide concise steps to clinicians at the point of care to guide antibiotic decision-making. At NYULH GN-BSI bundle was implemented in the electronic health record of the microbiology report. The purpose of this project was to evaluate the impact of the bundle on patient outcomes and prescribing.

**Methods:**

This was a multicenter, retrospective review of patients with GN-BSI in the pre-bundle group (preBG, 3/2019-2/2020) and post-bundle group (postBG, 3/2021 to 2/2022). The primary outcome was the composite of in-hospital mortality, infection-related readmission, and GN-BSI recurrence.

**Results:**

Out of 1097 patients screened, 225 patients met inclusion criteria n=101 in preBG, n=124 in postBG. Baseline characteristics were similar between groups, including Charlson Comorbidity Index [4 (IQR 3-6) vs. 4 (2-5); p=NS] and Modified PITT Bacteremia Score [1 (0-2) vs. 1 (0-2); p=NS]. The most common sources of GN-BSI were urinary (59 vs 59%, p=NS) and intra-abdominal (29 vs 23%, p=NS). *E. coli* (62 vs 61% p=NS) and *K. pneumoniae* (30 vs 21%, p=NS) were the most common organisms. There was no difference in the primary composite outcome (13 vs 7%; p=NS) and its individual components of in-hospital mortality, 30-day infection related readmission and GN-BSI recurrence (2 vs 0%, 10 vs 7%, 1 vs 1.6%). The median hospital length of stay (HLOS) from GN-BSI onset and days of therapy were also similar between the pre- and postBG [5 vs 5 days, p=NS; 14 vs 15 days, p=NS]. There was no difference between pre- and postBG in empiric vancomycin (VAN) initiation (57 vs 61%) and median VAN days (2 vs. 2 days). VAN discontinuation (DC) was done more frequently by the primary team in postBG group (67 vs. 38% preBG; p< 0.01). Numerically more patients were de-escalated in postBG (81 vs 73% preBG, p=NS) with 9% increase in the use of ampicillin/sulbactam or amoxicillin/clavulanate, p=0.043 and no difference in time to de-escalation. In postBG, de-escalation by the primary team and ASP increased numerically by 8.8%, p=NS and 4.4%, p=NS with a decrease in ID recommendations related to de-escalation by 13.6%, p=NS.

**Conclusion:**

GN-BSI bundle worked as a nudge-based tool to guide providers in VAN DC, de-escalation to more narrow antibiotics and decreasing amount of ID consults without negative impact on HLOS and patient outcomes.

**Disclosures:**

**All Authors**: No reported disclosures

